# Dynein axonemal heavy chain 8 promotes androgen receptor activity and associates with prostate cancer progression

**DOI:** 10.18632/oncotarget.10284

**Published:** 2016-06-24

**Authors:** Yu Wang, Russell J. Ledet, Keren Imberg-Kazdan, Susan K. Logan, Michael J. Garabedian

**Affiliations:** ^1^ Department of Urology, New York University School of Medicine, New York, NY, 10016, USA; ^2^ Department of Microbiology, New York University School of Medicine, New York, NY, 10016, USA; ^3^ Department of Biochemistry and Molecular Pharmacology, New York University School of Medicine, New York, NY, 10016, USA

**Keywords:** prostate cancer, DNAH8, androgen receptor, prognosis, metastasis

## Abstract

To gain insight into cellular factors regulating AR action that could promote castration resistant prostate cancer (CRPC), we performed a genome-wide RNAi screen for factors that promote ligand-independent AR transcriptional activity and integrated clinical databases for candidate genes that are positively associated with prostate cancer metastasis and recurrence. From this analysis, we identified *Dynein Axonemal Heavy Chain 8* (*DNAH8*) as an AR regulator that displayed higher mRNA expression in metastatic than in primary tumors, and showed high expression in patients with poor prognosis. Axonemal dyneins function in cellular motility, but the function of *DNAH8* in prostate cancer or other cell types has not been reported. *DNAH8* is on chromosome 6q21.2, a cancer-associated amplicon, and is primarily expressed in prostate and testis. Its expression is higher in primary tumors compared to normal prostate, and is further increased in metastatic prostate cancers. Patients expressing high levels of *DNAH8* have a greater risk of relapse and a poor prognosis after prostatectomy. Depletion of *DNAH8* in prostate cancer cells suppressed AR transcriptional activity and proliferation. Androgen treatment increased *DNAH8* mRNA expression, and AR bound the *DNAH8* promoter sequence indicating *DNAH8* is an AR target gene. Thus, *DNAH8* is a new regulator of AR associated with metastatic tumors and poor prognosis.

## INTRODUCTION

Prostate cancer growth relies on androgens and the androgen receptor (AR) pathway. Therefore, blocking this pathway through androgen deprivation and AR antagonists is the mainstay of prostate cancer treatment. However, prostate cancer patients receiving androgen deprivation therapy often relapse and develop castration-resistant prostate (CRPC), which still relies on the AR pathway for growth. Tumors at this advanced stage are highly proliferative and metastatic in nature, inevitably leading to fatality due to lack of effective therapies. The mechanism underlying the development of CRPC is still not fully understood, and assessing a patient's risk of tumor recurrence prior to androgen deprivation is a major challenge for the management of prostate cancer.

Growing evidence has shown that ligand-independent AR function in CRPC is regulated through a network of cell signaling pathways that are altered during disease progression [1–5]. Alteration in the expression or activity of AR regulatory factors may compensate for castration-induced AR inhibition and promote hormone-independent AR activity. Although recent studies have identified a few established cancer pathways regulating AR activity in CRPC, such as WNT and PI3K [6, 7], the landscape of molecular pathways controlling ligand-independent AR function and tumor growth remains undetermined.

To gain insight into the cellular factors controlling AR activity and potentially CRPC, we performed an unbiased, genome-wide siRNA screen for factors that reduced AR activity upon depletion using a *Drosophila* cell system. This was followed by functional analyses of homologous factors in human prostate cancer cells, revealing several new factors that regulate AR activity and prostate cancer cell proliferation [8].

In this current study, we combined clinical datasets containing genome-wide expression profiles and patient prognosis data with our RNAi screen candidates to reveal new AR co-regulators that promote AR activity during tumor progression. Using this approach, we discovered Dynein Axonemal Heavy Chain 8 (*DNAH8*) as an AR regulatory factor that is overexpressed in metastatic prostate cancer and predictive of poor prognosis. Using prostate cancer cell models, we investigated the regulation and crosstalk between AR and DNAH8. This revealed a feed-forward loop between AR and DNAH8, such that DNAH8 controls AR transcriptional activity and AR controls *DNAH8* expression. Thus, our integrated analysis revealed *DNAH8* as a putative high-priority therapeutic target and prognostic indicator in prostate cancer.

## RESULTS

### Genome-wide RNAi screen for CRPC-associated AR activators

**Figure 1:**
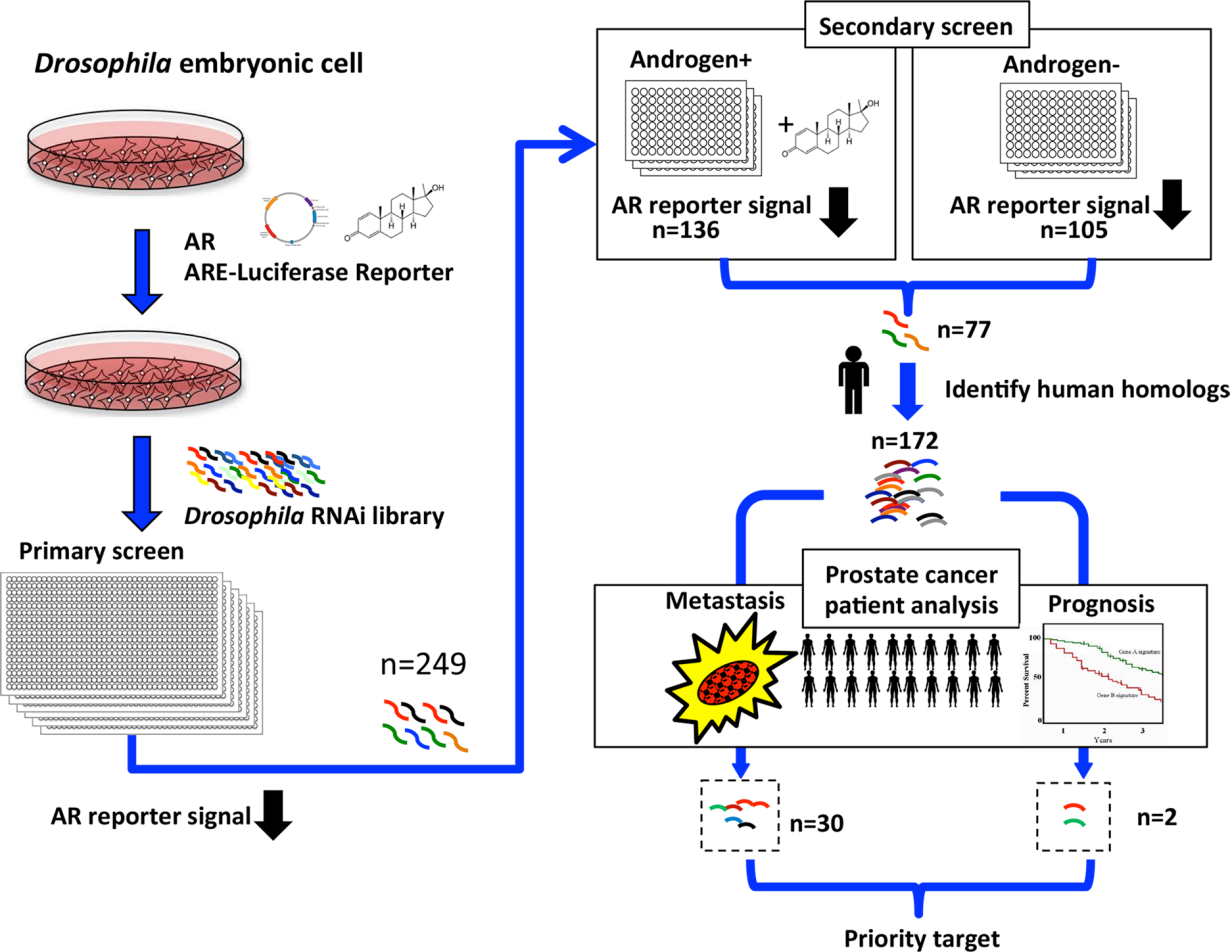
Selection scheme for clinically relevant AR regulators in prostate cancer Flowchart showing the RNAi screen designed to identify positive regulators of AR transcriptional activity. *Drosophila* S2 cells were transfected with AR and AR-responsive reporter construct, and AR-dependent transcriptional activity was quantified by luciferase activity in the presence of the synthetic androgen R1881. From the primary screen, 249 potential AR regulators were identified that reduced AR-dependent transcriptional activity when depleted. A secondary validation screen was performed, revealing 136 and 105 RNAis that reduced androgen-dependent (+Androgen) and androgen-independent (-Androgen) AR transcriptional activation. Of those, 77 reduced both androgen-dependent and -independent AR transcriptional activity. The 77 *Drosophila* genes represent 172 human homologs, of which 30 genes displayed higher mRNA expression in metastatic than in primary tumors, and two genes showed higher expression in patients with poor prognosis than in patients with good prognosis (*p* < 0.05).

To identify potential regulators underlying AR activation during CRPC progression, we combined gene expression data from clinical databases with our previous developed genome-wide RNAi screen [8] for cellular factors affecting AR-dependent transcriptional activity (Figure 1). The screen used a reconstituted human AR-mediated transcriptional readout in *Drosophila* S2 cells. From the screen, we identified 77 *Drosophila* genes whose RNAi depletion suppressed AR activity by over 50% in both the absence and presence of androgen (*p* < 0.05, Figure 1), indicating that these are likely candidates controlling AR-dependent gene expression under both normal and low levels of androgen.

To elucidate the biological relevance of these potential AR activators in human cancer, we used Ensemble 82 [9] to search for human homologs to the 77 *Drosophila* genes and identified 172 human genes. The higher number of human genes reflects the redundancy of the *Drosophila* genes in the human genome. We next interrogated publicly available genome-wide transcriptome datasets from the Gene Expression Omnibus (GEO) [10], cBioportal [11], and The Cancer Genome Atlas (TCGA) for changes in mRNA expression of the 172 genes during prostate cancer progression. We included data from cohorts that contained complete genome-wide expression profiles, detailed pathology information, and follow-up data. We excluded cultured cells and xenograft samples. The primary tumor cases were from radical prostatectomies without prior treatment to eliminate confounding effects of drug treatment on gene expression. Using these criteria, we analyzed mRNA expression data from 210 primary tumor samples from radical prostatectomy, in which 68 patients experienced relapse, and also interrogated mRNA expression profiles from another cohort of 44 metastatic cases [12]. Among the 172 candidate genes, 30 had significantly higher expression (*p* < 0.05) in metastatic compared to primary tumors (Figure 1 and Supplementary Figure S1), suggesting an association with prostate cancer progression. Many of these 30 genes, including *CCNA2* (Cyclin-A2*), MTA1* (Metastasis Associated 1), and *PAK4* (P21 Protein (Cdc42/Rac)-Activated Kinase 4), were previously reported to be associated with tumor metastasis and progression in various cancers [13–15]. This provided strong evidence for the efficacy of our selection strategy. We also identified two genes that have higher expression in patients with recurrent disease (*n* = 68) than in patients with relapse-free outcome (*n* = 142, *p* < 0.05, Figure 1 and Supplementary Figure S1). After cross-referencing the list of 30 progression-associated genes with the two recurrence-associated genes, we achieved only one common target: Dynein Axonemal Heavy Chain 8 (*DNAH8*) *8*, a gene without any previous report on its function.

### DNAH8 mRNA expression is associated with prostate cancer progression and poor prognosis

**Figure 2:**
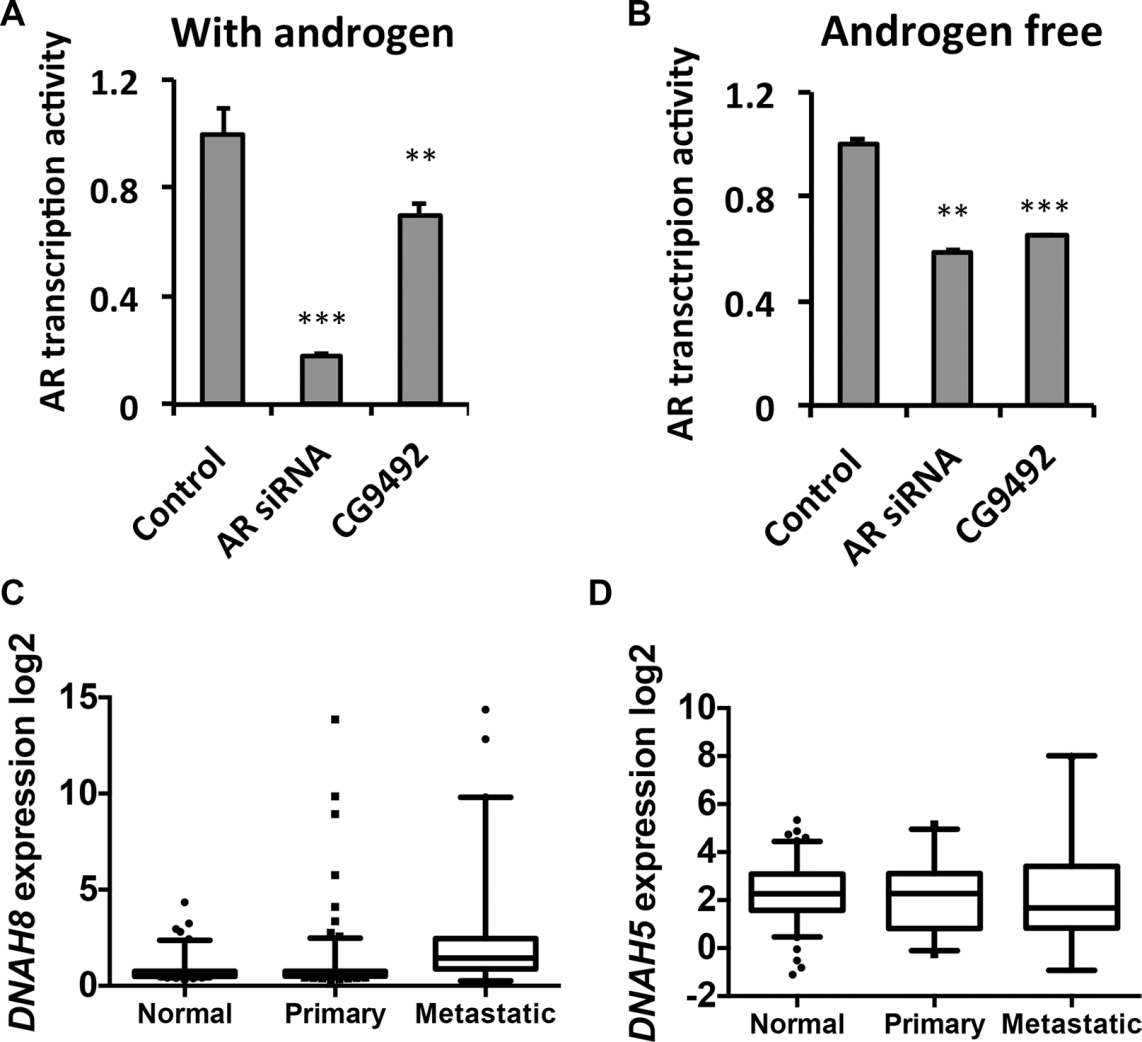
DNAH8 depletion decreases AR-mediated transcriptional activation and is associated with metastatic disease (**A)** and (**B**) *Drosophila* S2 cells were transfected with a human AR expression and ARE-luciferase reporter plasmids, along with a control scrambled siRNA (control), AR directed siRNA (AR siRNA), or an siRNA to *DNAH8* (CG9492), and luciferase activity was determined after 24 hr in the absence and presence of androgen. **p < 0.01, ***p < 0.001. Error-bars represent standard deviation of three independent experiments. **p < 0.01, ***p < 0.001. (**C**) Box and whisker plot of *DNAH8* mRNA expression in normal (*n* = 235) compared to primary (*n* = 235; *p* = 0.0049), and primary (*n* = 235) compared to metastatic (*n* = 44) prostate cancers (*p* < 0.0001). (**D**) DNAH5 does not show significantly different mRNA expression in normal prostate, primary tumors, and metastatic tumors (*p* = 0.78).

Consistent from our initial RNAi screen, depletion of the *Drosophila* homolog of *DNAH8* (*CG9492*) using RNAi significantly decreased AR transcriptional activity in the presence and absence of androgen in *Drosophila* S2 cells (Figure 2A and 2B). *CG9492* has homology to two genes in human genome, *DNAH8* and *DNAH5* (Dynein Axonemal Heavy Chain). *DNAH5* and *DNAH8* are homologous, displaying 60% sequence identity. To determine the potential relevance of *DNAH8* and *DNAH5* in human prostate cancer, we examined for differences in mRNA expression among normal, primary and metastatic samples from independent validation cohorts containing 235 normal prostate, 329 primary tumor and 59 metastatic tumor cases [16–18]. Whereas *DNAH8* mRNA expression was significantly increased in primary tumors compared with normal prostate (*p* = 0.0049), and was further up-regulated in metastatic tumors (*p* < 0.0001, Figure 2C), DNAH5 expression did not significantly differ among normal prostate, primary or metastatic prostate tumors (*p* = 0.78, Figure 2D). It should be noted that the mRNA expression of *DNAH8* and *AR* were not significantly correlated in the metastatic prostate cancer cases (*p* = 0.45. Supplementary Figure S2),suggesting that DNAH8 might be an independent prognostic indicator. Thus, the mRNA expression of *DNAH8*, but not *DNAH5*, is upregulated in metastasis relative to primary prostate tumors, suggesting a role for *DNAH8* in prostate cancer progression.

**Figure 3:**
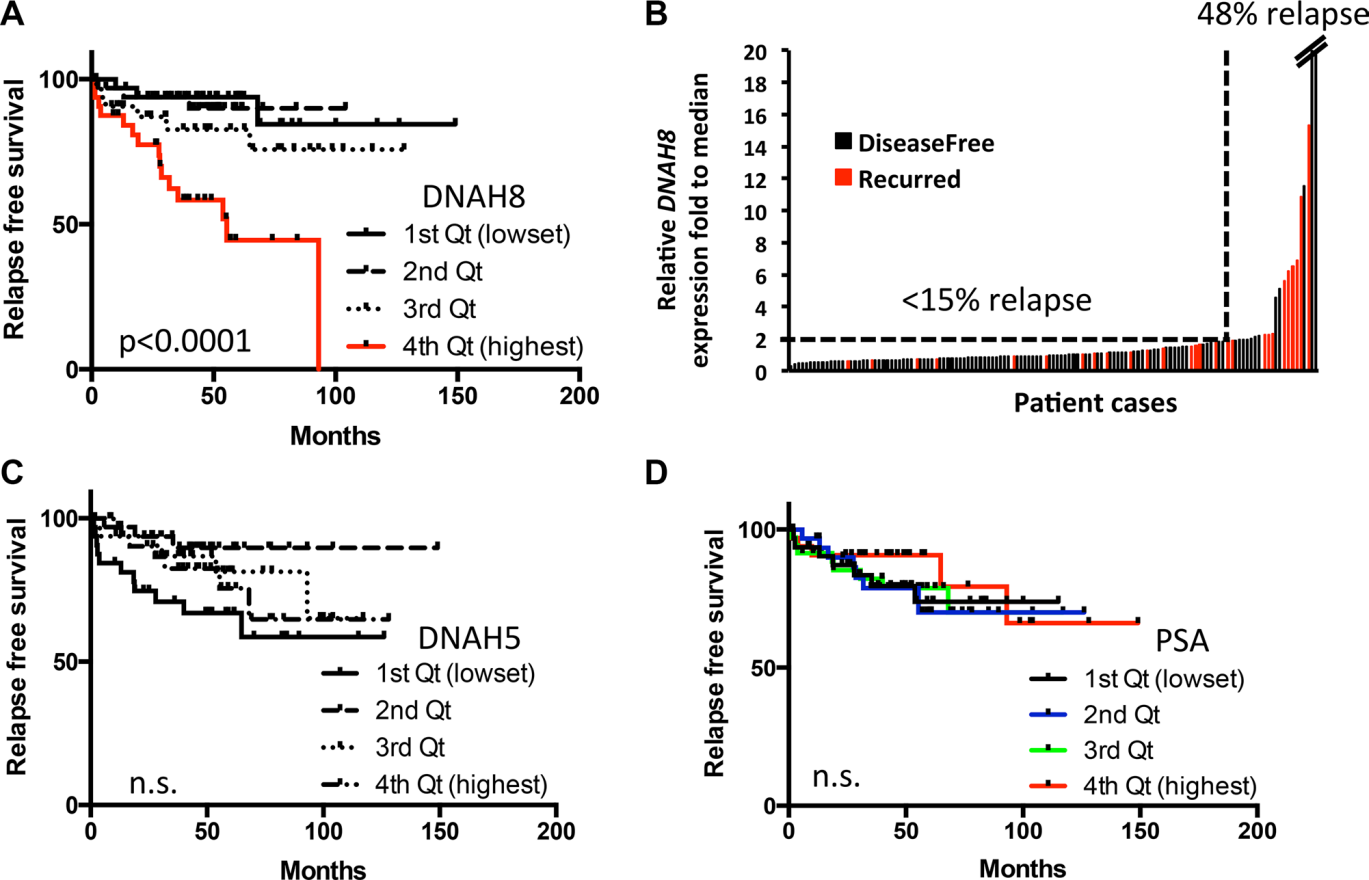
High DNAH8 mRNA expression is associated with reduced relapse free survival (**A**) Kaplan-Meier curves showing an association between *DNAH8* mRNA expression [4th quartile (Qt)] and relapse-free survival (*n* = 131). (**B**) Patients with the highest DNAH8 mRNA expression are at the greatest risk of disease relapse (*p* < 0.0005). (**C**) *DNAH5* or (**D**) PSA mRNA expression is not associated with relapse-free survival. n.s.:non-significant.

We next used retrospective analysis to determine whether *DNAH8* expression was associated with tumor recurrence after androgen deprivation. We chose a cohort of 131 patients who have prostate cancer Gleason scores ranging from 7 to 9 and have received radical prostatectomy. Patients were grouped into four quartiles according to the rank of mRNA expression for *DNAH8, DNAH5* or *PSA*. Patients in the highest quartile of *DNAH8* mRNA expression had the worse prognosis, with a median relapse-free survival (RFS) of 55.4 months (*p* < 0.0001, Figure 3A). This was in contrast to patients in the lowest three quartiles of *DNAH8* mRNA expression that had a good prognosis (median RFS not reached), and showed no difference in their incidence of relapse (*p* = 0.42, Figure 3A). We also found that *DNAH8* mRNA expression was not normally distributed (Shapiro-Wilk test, *p* < 2.2E-16), with a subgroup of patients having dramatically higher levels of *DNAH8* (Figure 3B). Of the patients with twice the median level of *DNAH8* mRNA expression, 48% (11/23) relapsed, whereas patients below this level had only 14.8% (16/108) relapse (Chi-square *p* = 0.0004, Figure 3B). We did not find a significance association in the mRNA expression of *DNAH5* or *PSA* in primary tumors with RFS (*p* = 0.87 and 0.09 prospectively, Figure 3C and 3D). This indicates a strong and specific association between *DNAH8* mRNA expression and prostate cancer progression.

### Characterization of human DNAH8

**Figure 4:**
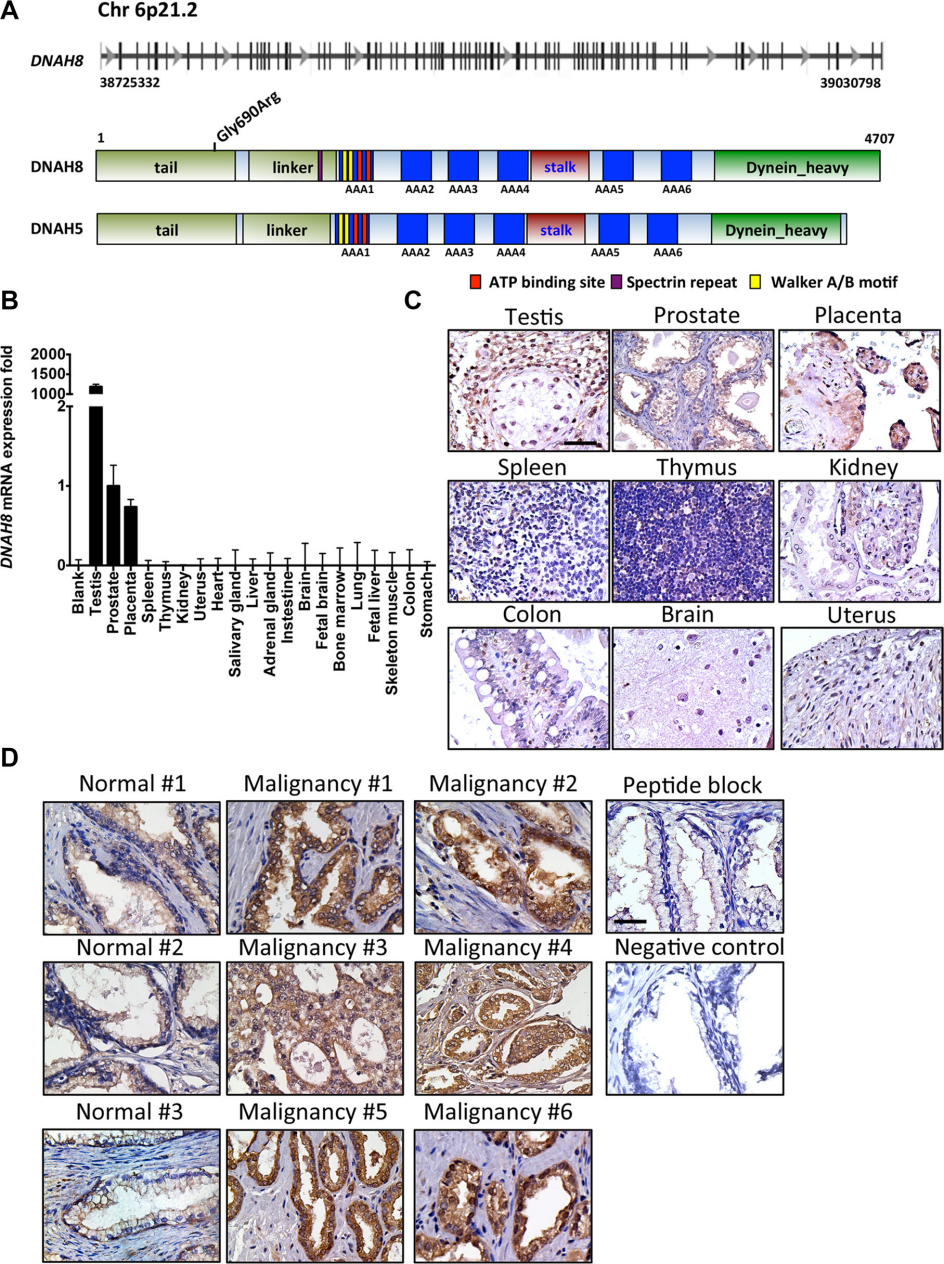
DNAH8 mRNA expression and protein abundance in normal human tissues and prostate cancer (**A**) Schematic representation of the DNAH8 gene and protein architecture. Top panel shows the *DNAH8* mRNA and intron/exon structure, and the bottom panel displays the DNAH8 protein and its predicted functional domains. (**B**) *DNAH8* mRNA expression from 20 different normal human tissues by qRT-PCR. (**C**) Immunohistochemistry of DNAH8 protein expression from different normal human tissues. Scale bar = 60 μm. (**D**) Representative images of DNAH8 protein expression in normal prostate (*n* = 4) and prostate cancer (*n* = 6) by immunohistochemistry. Note the increased immunoreactivity (seen as brown staining) in prostate cancer relative to normal prostate tissue. Scale bar = 60 μm.

**Figure 5:**
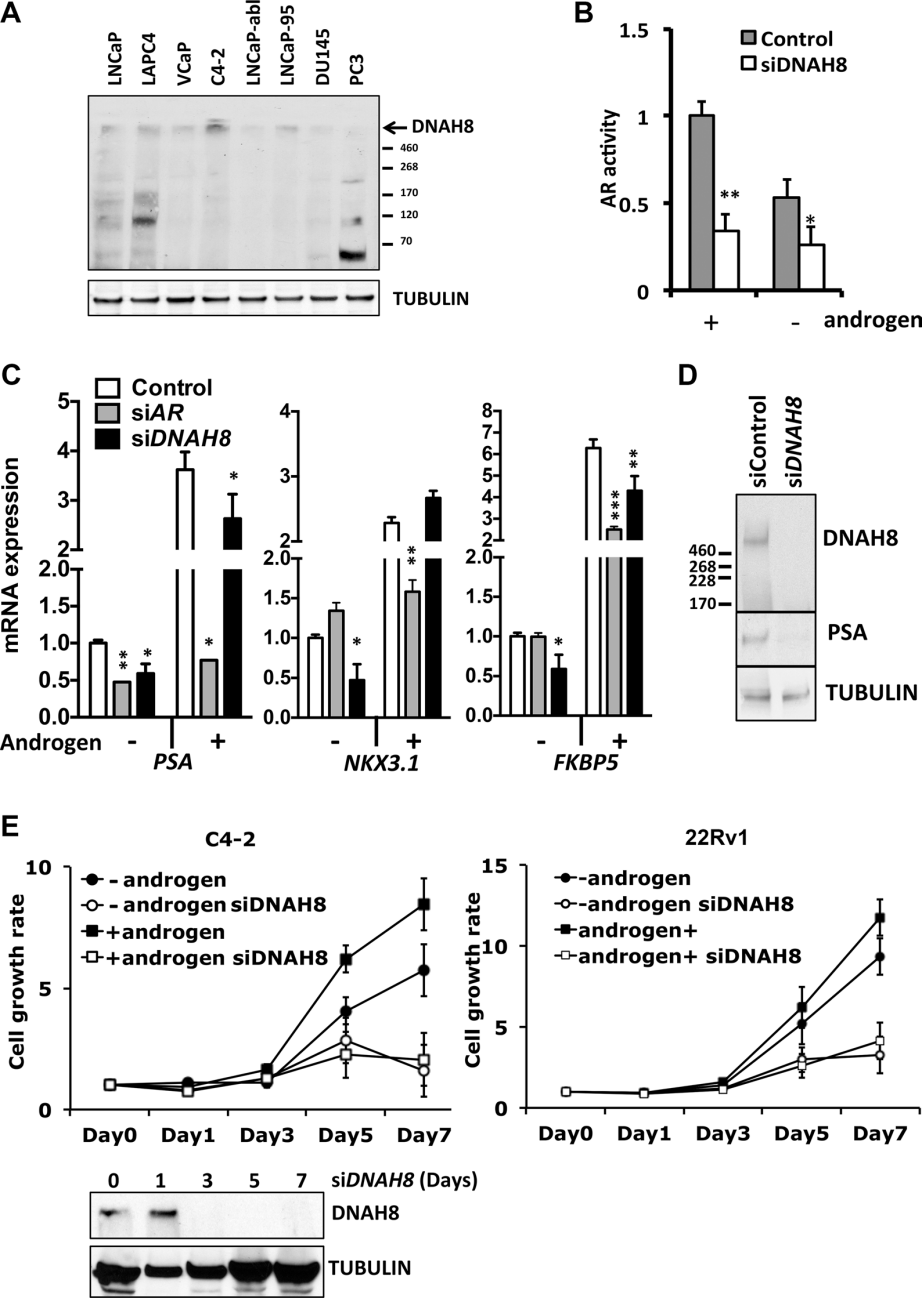
DNAH8 protein expression in cultured prostate cancer cell lines, and reduced AR-dependent transcriptional activity and proliferation upon DHAH8 depletion (**A**) Western blot analysis shows DNAH8 protein abundance in multiple AR expressing (LNCaP, LAPC4, VCaP, C4-2,LNCaP-abl, and LNCaP-95) and non-AR expressing (DU145 and PC3) prostate cancer cell lines. (**B**) LNCaP cells were transfected with an AR luciferase reporter gene along with control or DNAH8 siRNA and luciferase activity determine after 72 hr in the presence of complete androgen containing media (grey bars) as well as in androgen-free media (white bars). **p < 0.01, *p < 0.05. (**C**) C4-2 cells were transfected with control, AR siRNA, or DNAH8 siRNA. After four days of culture in androgen-negative or –positive media, total RNA was extracted from cell lysate for reverse transcript. AR-target gene (*PSA*, *NKX3*.1 and *BKBP5*) expression was quantified by real-time PCR. ***p < 0.001, **p < 0.01, *p < 0.05. (**D**) Western blot analysis shows that *DNAH8* knockdown effectively depletes DNAH8 protein expression and decreased PSA protein abundance in C4-2 cells. (**E**) C4-2 and 22Rv1 cells were transfected with control, or DNAH8 siRNA and cell proliferation was measured in complete (+androgen) media or androgen-free media. Western blots (bottom) showed DNAH8 knockdown by siRNA during the 7-day period.

*DNAH8* is located at 6p21.2. The gene spans 315 kb with 95 exons and a predicted transcript length of ~15 kb (Figure 4A). The 6p21.2 region resides in an amplicon that is significantly associated with many human malignancies including prostate cancer [19]. The *DNAH8* mRNA encodes a predicted protein of 4,707 amino acids (NCBI NP_001193856) and a molecular mass of ~550 kDa (Figure 5). Since there are no reports on the structure or biological function of DNAH8, we used NCBI Conserved Domain Architecture Retrieval Tool and Pyre2 [20] to predict the potential functional domains of DNAH8. DNAH8 shares a number of conserved domains with members of the dynein axonemal heavy chain family. It contains N-terminal tail and linker domains, which include a dynein heavy chain N-terminal (DHC_N1) and DHC_N2 motifs potentially involved in dimer formation (Figure 4A). There are also six predicted AAA+ (ATPases Associated with a wide variety of cellular Activities) motifs in DNAH8. The identification of Walker A and Walker B motifs and two ATP-binding sites further suggests its function as an ATPase. There is also a microtubule-binding stalk of dynein motor (MT) domain and a dynein heavy chain domain at its C-terminus, consistent with microtubule-binding function.

Interrogation of 1172 cases (HGVST1) from genome-wide association studies (GWAS) [21, 22] identified a single nucleotide polymorphism (SNP) in the protein-coding region of *DNAH8* (rs173854, Gly690Arg) that is located in DHC_N1 motif and is positively associated with the incidence of prostate cancer (*p*= 0.0018). This further supports a link between *DNAH8* and prostate cancer. Whether this change resulted in a gain or loss of function is unknown.

The strong association between *DNAH8* and human prostate cancer prompted us to further investigate its expression in normal human tissues. We examined *DNAH8* mRNA expression from 20 normal human tissues by qRT-PCR. *DNAH8* mRNA was primarily expressed in testis and prostate in males and placenta in females (Figure 4B). Immunohistochemistry (IHC) using a DNAH8-specific antibody confirmed this tissue specific mRNA expression as positive staining was only observed in testis, prostate, and placenta (Figure 4C).

Next, we examined DNAH8 protein abundance from radical prostatectomy specimens. DNAH8 protein was observed in prostate epithelial, but not stromal cells. DNAH8 protein was predominantly expressed in the cytoplasm. Malignant lesions had dramatically increased DNAH8 staining when compared with adjacent normal prostate tissue (Figure 4D), further suggesting an association of DNAH8 with tumor development and progression that is consistent with the mRNA expression analysis.

### DNAH8 activates AR function and promotes the proliferation of prostate cancer cell lines

To explore DNAH8 function in prostate cancer and its regulatory effect on the AR pathway, we examined DNAH8 protein expression from eight patient-derived prostate cancer cell lines (Figure 5A). These included AR-expressing, androgen-dependent LNCaP, VCaP, LAPC4, and androgen-independent LNCaP-abl, C4-2, LNCaP-95, as well as AR-deficient DU145 and PC3 cells. DNAH8 was detected as a single large molecular weight band that exceeded the largest 460 kDa marker on the gel in a majority of the cell lines (Figure 5A), consistent with its predicted size of ~550 kDa. In some cells (e.g. PC3), we saw additional lower molecular weight bands. We do not yet know if these represent alternatively spliced products or translational start site variants, degradation products or cross reacting proteins. Nevertheless, DNAH8 protein abundance of the predicted size appeared highest in AR-expressing LNCaP and C4-2 cells, and lowest in non-AR-expressing PC3 and DU145. This suggests that DNAH8 expression might be AR regulated.

To test the impact of *DNAH8* on AR-dependent gene expression, we depleted DNAH8 by siRNA and examined AR transcriptional activity. Depletion of DNAH8 in LNCaP cells decreased AR-dependent transcriptional activity of a reporter gene under both androgen-dependent and androgen-independent conditions (Figure 5B), consistent with our findings upon *CG9492* (the *Drosophila DNAH8* homolog) depletion in the *Drosophila* cell model (Figure 2). DNAH8 depletion also affected endogenous AR-target gene expression. Expression of *PSA*, *NKX3.1* and *FKBP5* mRNA was reduced upon *DNAH8* siRNA depletion in the absence androgens in C4-2 cells. Similarly, DNAH8 depletion decreased androgen stimulated *PSA* and *FKBP5* mRNA expression, but had little effect on *NKX3.1* (Figure 5C). Depletion of *DNAH8* in C4-2 cells also reduced PSA protein abundance (Figure 5D). This suggests that *DNAH8* affects both androgen-independent and -dependent AR-mediated transcriptional activity.

To determine whether DNAH8 affects androgen-dependent and -independent proliferation of prostate cancer cells, we depleted *DNAH8* by siRNA in C4-2 (full-length AR) and 22Rv1 (full-length AR and AR splicing variant) cells, in both the absence and presence of androgens (Figure 5E).While these cell lines can proliferate in the absence of androgens (Figure 5C), they are AR-dependent as AR depletion suppressed cell proliferation (Supplementary Figure S3). DNAH8 depletion reduced the proliferation of C4-2 and 22Rv1 cells (Figure 5E, lower panel) under androgen-free and androgen-containing conditions (Figure 5E). Therefore, DNAH8 affects both androgen-dependent and -independent prostate cancer cell proliferation, in part by controlling AR activity.

**Figure 6:**
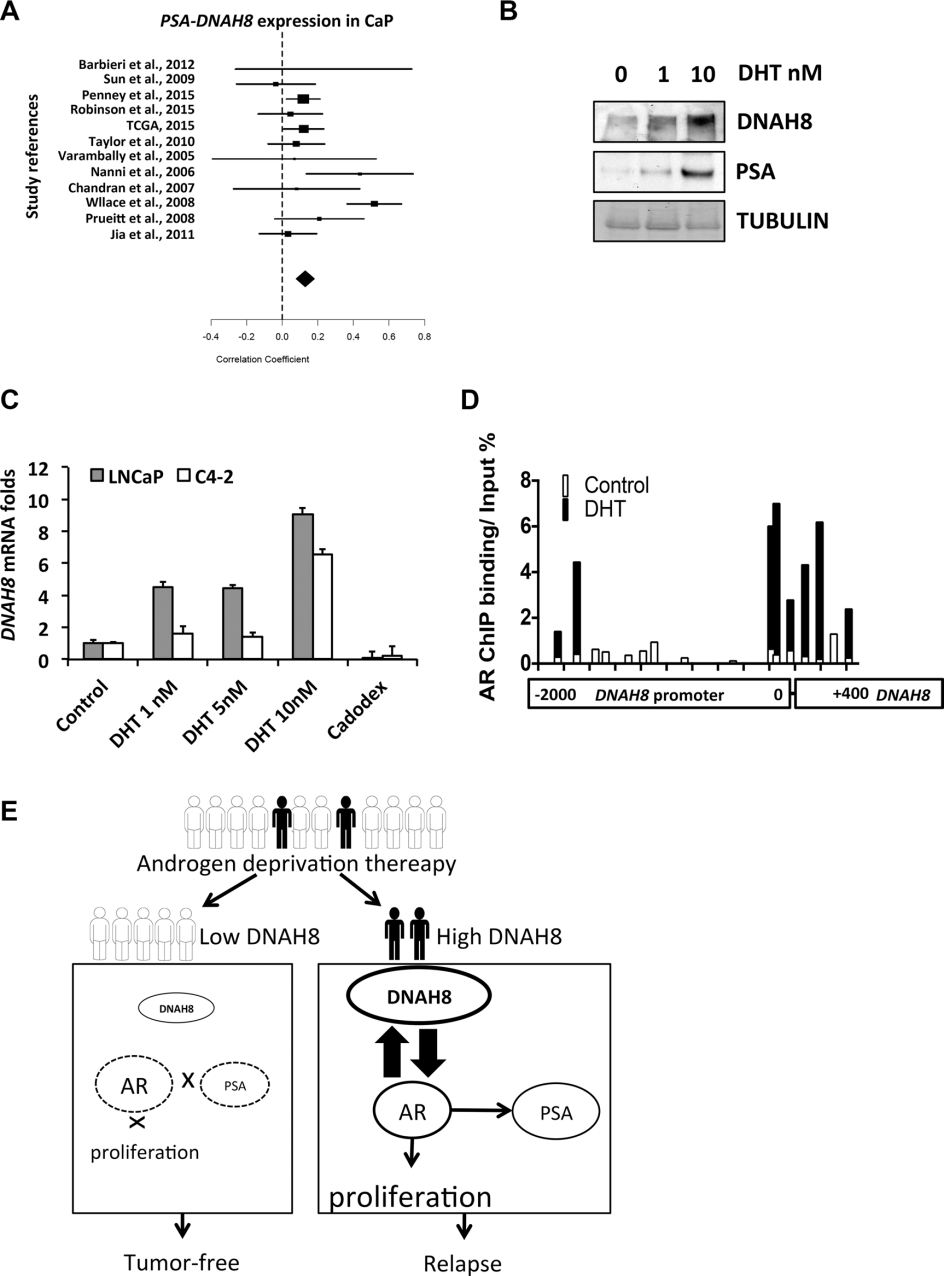
AR promotes *DNAH8* transcription in prostate cancer cells (**A**) Meta-analysis from 1476 prostate cancer cases shown a positive correlation between *DNAH8* and *PSA* expression in prostate cancer (correlation coefficient 0.13, upper limit 0.18, lower limit 0.08, *p* < 0.05). (**B**) C4-2 cells were treated with vehicle or the indicated concentrations of DHT for 48 hours and western blot for DNAH8 and PSA was performed. (**C**) LNCaP and C4-2 cells in complete media were treated as above with vehicle, DHT, or the AR antagonist Casodex (10 μM). After 48 hr, cell RNA was harvested for q-RT-PCR to measure *DNAH8* expression levels. (**D**) AR binds to the *DNAH8* promoter. C4-2 cells in compete-media were treated with control or 10 nM DHT for 24 hr, and a ChIP assay for AR was performed. Primer pairs that span the −2000 to +400 region of the DNAH8 promoter were used to assess AR recruitment to the DNAH8 promoter. AR occupancy in the absence (white bars) and presence (black bars) of DHT treatment is shown. Values are normalized to input DNA. (**E**) Schematic showing DNAH8 regulates AR in prostate cancer after androgen deprivation.

To examine if *DNAH8* expression coincides with AR activity in prostate cancer patients, we took a meta-analysis approach and examined the mRNA expression patterns of *DNAH8* and *PSA* as a surrogate for active AR signaling. We examined expression data of 1476 patient cases from 12 publicly available datasets [12, 16, 17, 24–30]. If DNAH8 promoted AR activity then a positive association between *DNAH8* and *PSA* mRNA expression would be observed. In fact, our analysis revealed a positive association between *DNAH8* and *PSA* mRNA expression in prostate cancer (correlation coefficient 0.13, upper limit 0.18, lower limit 0.08, Figure 6A). *DNAH8* was also positively associated with *NKX3.1* (correlation coefficient 0.15, upper limit 0.23, lower limit 0.08, and *p* < 0.05) and *FKBP5* (correlation coefficient 0.08, upper limit 0.15, lower limit 0.01, and *p* < 0.05) expression in prostate cancer (Supplementary Figure S4). This is consistent with *DNAH8* mRNA expression being associated with AR activity.

Another potential interpretation of the meta-analysis is that the expression of *DNAH8* and *PSA* could be co-regulated by AR in prostate. A recent AR ChIP-seq study in prostate cancer [5] revealed AR occupancy at the *DNAH8* promoter, suggesting that *DNAH8* expression has the potential to be regulated by AR. To test this, we treated C4-2 cells with increasing amounts of DHT and examined DNAH8 and PSA protein abundance. We found that both DNAH8 and PSA levels were enhanced by DHT treatment (Figure 6B). To determine whether androgen-induced AR activation promotes *DNAH8* transcription, we treated LNCaP and C4-2 cells with DHT or the AR antagonist bicalutamide (casodex). As expected, DHT stimulated the *PSA* and *NKX3.1* transcription in both cells line while casodex suppressed the expression of both genes (Supplementary Figure S5). DHT treatment increased *DNAH8* mRNA levels in a dose-dependent manner in both cell lines, and casodex suppressed the androgen-induced *DNAH8* expression (Figure 6C). In addition, AR overexpression resulted in a moderate DNAH8 expression increase in multiple prostate cancer cell lines (Supplementary Figure S6). Together these data suggest that AR promotes *DNAH8* mRNA and protein expression.

We next examined whether AR could directly stimulate the *DHAH8* promoter in a hormone-dependent manner in C4-2 cells by interrogating AR occupancy on the promoter and regulator region of *DNAH8* locus by ChIP. In C4-2 cells AR does respond to androgen treatment [31], and AR was recruited to the ARE in the *PSA* promoters upon DHT stimulation (Supplementary Figure S7). We identified two regions in the *DNAH8* promoter that showed AR recruitment upon DHT treatment. The first region is near the *DNAH8* transcription start site and the second is located further upstream in −1700 to −1860 region (Figure 6D). This suggests that *DNAH8* is a direct AR target gene and that AR activation promotes *DNAH8* transcription.

## DISCUSSION

Recent studies comparing primary to metastatic prostate tumors have revealed several signaling pathways that promote AR activation under androgen-deprivation [1–5]. For example, loss of *PTEN* in prostate cancer led to hyper-activation of AKT, which induced AR phosphorylation and androgen-independent activation [2, 32]. Activation of nuclear beta-catenin enhanced AR transcriptional activity by serving as an AR co-activator [1, 33]. This has not only led to a better understanding of the mechanisms underlying AR activation in the progression of prostate cancer, but has also provided targets for new therapeutic interventions [34].

To continue to elucidate the factors and pathways that participate in AR signaling in prostate cancer, we performed a genome wide RNAi screen to identify new AR regulators that contribute to AR activity and potentially CRPC [8]. Although we have previously reported several new AR co-activators from this screen, including HIPK2 and MED19, these factors were selected empirically based on their potential as drug targets or because of the magnitude of their effect on AR transcriptional activity and cell proliferation upon depletion.

To distinguish the most clinically relevant AR coregulators in CRPC, we revaluated the “hits” from our RNAi screen based on mRNA expression data linked to clinical outcomes. In doing so, we identified 30 genes from the RNAi screen with significantly increased mRNA expression in metastatic compared to primary tumors.In addition, we found increased expression of two genes in patients with recurrent *vs.* non-recurrent tumors, including *DNAH8*, which was in both categories, leading us to study its function as a high value AR coactivator. We also plan in future studies to analyze data from additional cohorts to validate the association between DNAH8 and prostate cancer patient prognosis and metastasis.

Consistent with a role for DNAH8 in promoting AR activity and prostate cancer progression, *DNAH8* resided in a cancer-associated amplicon, and contained a coding SNP in prostate cancer [19]. It was primarily expressed in normal prostate and exhibited higher expression in prostate cancer, and was positively associated with metastatic prostate tumors and correlated with disease recurrence.

Although there are no reports chronicling DNAH8′s action, phylogenetic sequence analysis suggested that DNAH8 is an axonemal dynein and likely an outer-arm axonemal dynein (data not shown). Here we report that DNAH8 functions in prostate cancer and affects AR signaling. Depletion of DHAH8 reduced AR transcriptional activation and cellular proliferation, suggesting a link between DNAH8 and AR activity. Consistent with this idea, meta-analysis revealed that *DNAH8* and AR-target gene expression were positively associated in prostate caner. It is interesting that *DNAH8*, but not *PSA* mRNA expression in primary tumors was associated with tumor recurrence (Figure 3). This suggests that factors in addition to *DNAH8* regulate AR-mediated gene expression during prostate cancer initiation and progression, and is consistent with multiple AR regulatory factors being identified in our RNAi screen.

Whereas DNAH8 protein abundance appeared higher in AR-expressing compared to non-AR-expressing prostate cancer cells lines (Figure 5A), this paralleled androgen-dependent *DNAH8* mRNA expression and AR recruitment to the *DNAH8* promoter. Thus AR and DNAH8 appear to form a positive feed forward loop with AR promoting *DNAH8* expression and *DNAH8* expression enhancing AR activity (Figure 6E). We also found that the *DNAH8* gene is amplified in metastatic prostate cancer (data not shown), consistent with elevated *DNAH8* mRNA expression in metastatic disease. Therefore, *DNAH8* copy number amplification, in concert with or independent of AR (Supplementary Figure S2), could promote *DNAH8* expression in advanced prostate cancer.

How DNAH8 controls AR activity remains unknown. *DNAH8* shares greater homology with axonemal (61%) as compared cytoplasmic dyneins (25%). Axonemal dyneins are involved in cilium or flagellum-dependent motility [35]. While this might suggest a link between DNAH8 and cell movement, it remains unclear how this might impact AR transcriptional activity, given there are no previous reports for axonemal dyneins' regulating AR activity. Although non-motile primary cilia have a cargo-transporting dynein similar to cytoplasmic dynein, they do not possess axonemal dynein motors, which drive movement of motile cilia and flagella. Therefore, signaling via primary cilia by DNAH8 is unlikely.

Cytoplasmic dynein binds cargo and transports them to various cellular locations. In fact, cytoplasmic dynein interacts with AR and promotes AR trafficking into the nucleus [37]. Disruption of this interaction dramatically reduced hormone-induced AR nuclear accumulation [38]. It is possible that upon increased *DNAH8* expression during prostate cancer progression, DNAH8 could increase AR and/or AR cofactor nuclear accumulation and foster androgen-independent AR activation. Alternatively, DNAH8 might function as an AR co-activator independent of it's axonemal or putative cytoplasmic dynein function and perhaps even work in the nucleus, bind to AR and serve as a more traditional AR co-activator. This later idea is supported by nuclear DNAH8 staining in malignant, but not in non-neoplastic prostate cases (Figure 4D). Further studies on the mechanism of DNAH8-mediated AR activity will be required to elucidate the role DNAH8 plays in promoting ligand-independent AR activation in prostate cancer. In summary, by combining clinical data and bioinformatic analyses with data from a genome-wide RNAi screen for AR coregulators, we have identified *DNAH8* as a potential new AR activator whose expression is associated with metastasis and poor prognosis (Figure 6E).

## MATERIALS AND METHODS

### Cell culture and human specimen

Human prostate cancer cell lines were purchased from ATCC (LNCaP, VCaP, LNPC4, PC3, and DU145), and Urco (C4-2), and were maintained in RPMI1640, 10% FBS, 1% Penicillin-Streptomycin (complete media). LNCaP-abl (gift from Dr. Culig, Medical University of Innsbruck, Austria) and LNCaP-95 (gift from Dr. Ru, Johns Hopkins University, USA) were cultured in phenol read-free RPMI1640, 10% charcoal-stripped FBS, 1% Penicillin-Streptomycin (androgen-free media). All experiments in LNCaP and C4-2 cells were conducted in complete-media, unless mentioned otherwise. Cells were tested for mycoplasma and found to be negative. Cells were authenticated by short tandem repeat (STR) profiling (Genetica, Burlington, NC). Dihydrotestosterone (DHT) was purchased from Sigma and bicalutamide was purchased from MedKoo (Chapel Hill, NC).

### RNAi screen

Primary RNAi screen in *Drosophila* cells to identify potential AR regulators has been previously reported [8]. From this data we identified 77 *Drosophila* genes whose RNAi knockdown attenuated AR transcriptional activity in both the absence and presence of androgens (> 1.5 fold, *p* < 0.05). Using Ensemble 82 [9], we identified the homologous human genes. The human genes were then analyzed *in silico* for changes in mRNA expression from normal, primary and metastatic prostate tumor. The *siGC9492* in *Drosophila* and *siDNAH8* in human cell lines (pool of three different siRNAs) were from Santa Cruz Biotechnology (Supplementary Table S1).

### *In silico* analysis of candidate gene expression in prostate cancer

A search through The Cancer Genome Atlas (TCGA) and Gene Expression Omnibus (GEO), and cBioportal was performed to identify publicly available whole transcriptome datasets using non-cultured prostate carcinoma specimen derived from patients. Cases from microarray data were limited to the Affymetrix HGU133Plus2.0 (Santa Clara, California) GPL570, GPL93, GPL96, and GPL19370, which contain the probes for the *DNAH8* gene. We excluded data from patients who received prior or ongoing treatment before sample collection. Only datasets contain more than 20 cases were included. For candidate gene screening, we identified 254 tumor cases with tumor relapse data, and the 172 candidate genes expression profiles were extracted to compare metastatic tumors vs. primary tumors and to compare cases with relapse-free outcome vs. cases with tumor recurrence. We also identified a total of 224 normal prostate, 325 primary prostate tumor, and 44 metastatic tumor cases to analyze the expression of *DNAH8* and *DNAH5*. For retrospective analysis, we identified 131 primary tumor cases with comprehensive pathology and follow-up data, and analyzed the association of *DNAH8*, *DNAH5* and *PSA* with relapse-free survival. We also examined 1476 prostate cancer cases to analyze the correlation between *DNAH8* and *PSA* expression by R version 2.10.0 [39].

### Statistical analysis

AR reporter signal in luciferase assay and *DNAH8* gene expression in qRT-PCR were analyzed by unpaired *t*-test. *DNAH8* and *DNAH5* gene expression comparisons in normal prostate vs. primary tumor, and primary vs. metastatic tumor were analyzed by the Mann-Whitney test. Gene expression among these three groups was analyzed by the Kruskal–Wallis test. Association between *DNAH8* expression and tumor recurrence was analyzed by the Chi-square test. Gene expression association with relapse-free survival was accessed by the Kaplan-Meier analysis. *DNAH8* SNP analysis used genome-wide association study (GWAS) database. Statistical analysis was performed in Prism version 6.0 (GraphPad Software, La Jolla, CA), and SPSS version 13.0 (SPSS, Inc., Chicago, IL). All experiments were performed three times from independent biological replicates and the error bars represent the standard deviation with significance calculated by nonparametric *t*-test.

### Cell proliferation assay

Cell proliferation was determined using the MTT assay. C4-2 cells were transfected with control siRNA or anti-DNAH8 siRNA (Santa Cruz Biotechnology). Fifteen thousand cells were seeded in 24-well plates 24 hr before the “Day 0” point was measured. MTT assay was conducted on days 0, 1, 3, 5 and 7. Each well was incubated with 25 μL of 5 mg/mL MTT for 1 hour in a CO_2_ incubator at 37°C. The media was aspirated and 0.5 mL dimethyl sulfoxide (DMSO) was added per well. After a 10-minute incubation on shaker, 200 μL per well were transferred to a 96-well plate and proliferation rates were measured by colorimetric assay of formazan intensity in a plate reader at 560 nm.

### Western blotting

Western blotting was performed as described elsewhere [40]. Rabbit polyclonal anti-DNAH8 antibodies were purchased from Santa Cruz Biotechnology (sc-7350) for western blot and from ATLAS (HPA028447, Stockholm, Sweden) for IHC. Mouse monoclonal anti-alpha-Tubulin (T6199), anti-PSA (SAB1303590) antibodies were purchased from Sigma-Aldrich, St. Louis, MO.

### Quantitative reverse transcription-polymerase chain reaction (qRT-PCR)

Total RNA from human tissues panel representing 20 different organs, representing a mixture from multiple human specimens, (Clontech, Mountain View, CA) or prostate cancer cells was reversely transcribed to synthesize cDNA and gene expression was quantified by real-time PCR using SYBR Green (Applied Biosystem, Foster City, CA). Expression of RPL-19 was used as the internal control. Primer sequences are in Supplementary Table S2.

### Immunohistochemistry

FDA-grade human tissue microarray is from US Biomatrix (Rockville, MD). Patient radical prostatectomy specimens were from New York University Medical Center, NY. Tissues slides were rehydrated in xylene and a series of graded ethanol. Antigen retrieval was performed with 0.01 M citrate buffer at pH 6.0 for 20 minutes at 95°C. Slides were allowed to cool for another 30 minutes, followed by sequential rinsing in phosphate-buffered saline with 0.01% Triton X (PBS-T). Endogenous peroxidase activity was quenched by incubation in PBS-T containing 3% hydrogen peroxide. Each incubation step was carried out at room temperature and was followed by three sequential washes in PBS-T. Sections were incubated in 5% goat serum albumin in RT for one hour before rabbit polyclonal anti-DNAH8 antibody (Sigma-Aldrich) for overnight at 4°C. The next day, slides were washed with PBS-T three times and were incubated with biotinylated secondary antibody for 1 hour, peroxidase-labeled streptavidin (Vectastain system, Vector Laboratories) for 1 hour, and diaminobenzidine substrate for peroxidase-based immunohistochemistry (Cardassian DAB Chromogen, Biocare Medical). Slides were counter-stained with hematoxylin (Vector Laboratories) and dehydrated before mounted.

### Chromatin immunoprecipitation (ChIP) assay

C4-2 cells in 100 mm dishes were cultured overnight in androgen-free media. After 24 hrs of vehicle or DHT (10 nM) treatment, cells were harvested for ChIP assay using the EZ ChIP Assay Kit (Millipore) per manufacture instruction. Anti-AR antibody (2 μg) from Santa Cruz (AR441) was incubated with the lysates for 24 hr to precipitate AR. DNA associated with AR under androgen-free control, DHT treated, IgG control and 10% chromatin input were subjected to quantitative PCR with overlapping primer sets covering −2000 to +400 region of the *DNAH8* promoter (Supplementary Table S2). Each primer set amplification from the AR pull down was subtracted from the IgG control and then normalized to percent input.

## SUPPLEMENTARY MATERIALS FIGURES AND TABLES


