# Supplementary material for: Dynein axonemal heavy chain 8 promotes androgen receptor activity and associates with prostate cancer progression
Source: Oncotarget. 2016 Jun 24;7(31):49268–80. doi: 10.18632/oncotarget.10284 (PMC5226506; doi:10.18632/oncotarget.10284)
Supplement: Supplementary file 1 [file oncotarget-07-49268-s001.pdf]

Dynein axonemal heavy chain 8 promotes androgen receptor activity and associates with prostate cancer progression

Supplementary Materials

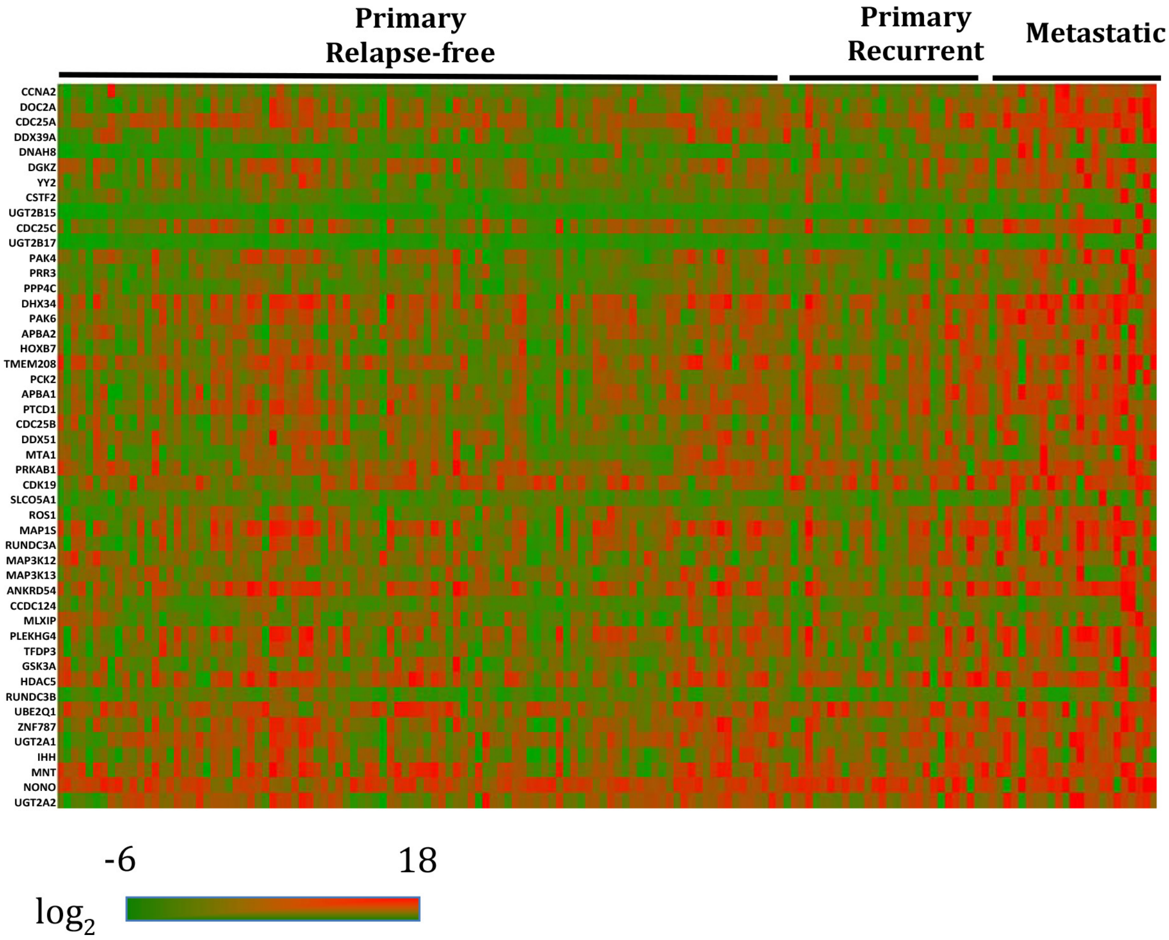

Supplementary Figure S1: Genome wide transcriptome data were extracted from 210 primary tumor samples from radical prostatectomy, in which 68 patients experienced relapse. We also recruited additional 44 long distance metastasis cases. Among the 172 candidate genes from our RNAi screen, 30 genes have significantly higher expression ($p < 0.05$) in metastatic tumors compared with primary tumors. Two genes have higher expression in patients with recurrent disease ($n = 68$) than in patients with relapse-free outcome ($n = 142$, $p < 0.05$). Heat map showing gene expression profiles of these 31 genes in primary tumors with relapse-free out come ($n = 142$), primary tumors with recurrence event ($n = 68$), and metastatic tumors ($n = 44$). Genes are ranked by p value with smallest on top. Red represents higher expression and green displays lower expression.

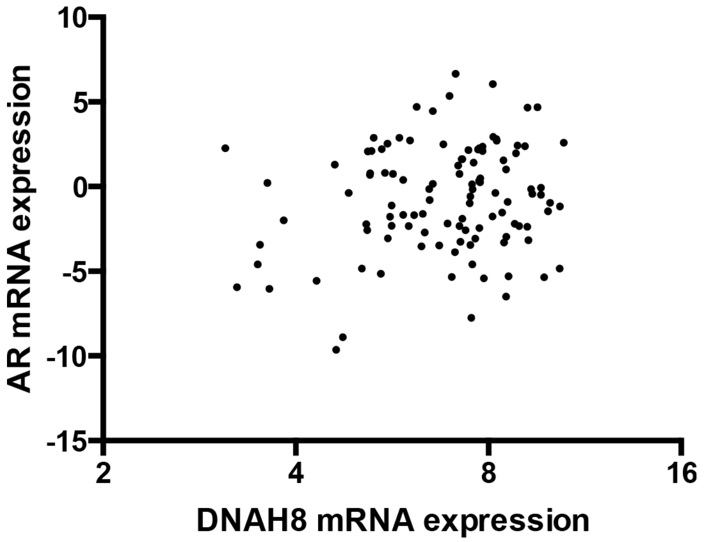

Supplementary Figure S2: AR and DNAH8 mRNA levels are not significantly correlated in metastatic prostate tumors. The gene expression profiles were extracted from 125 metastatic tumors. The expression of these two genes are not significantly correlated ($p = 0.45$).

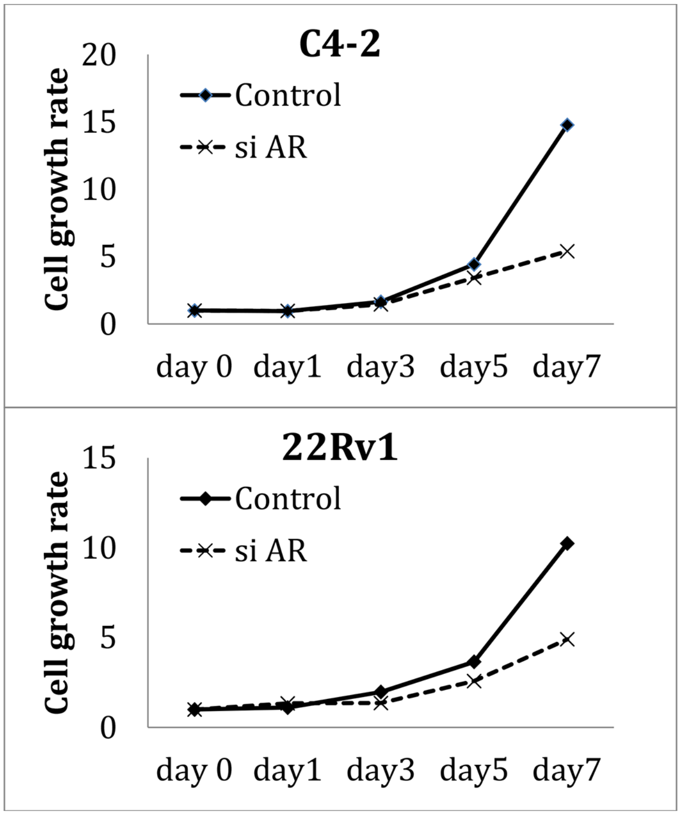

Supplementary Figure S3: AR depletion suppressed androgen-independent, AR-dependent C4-2 and 22Rv1 cells growth. Cell were transfected with AR siRNA and cell proliferation was measured by MTT assay for 7 days.

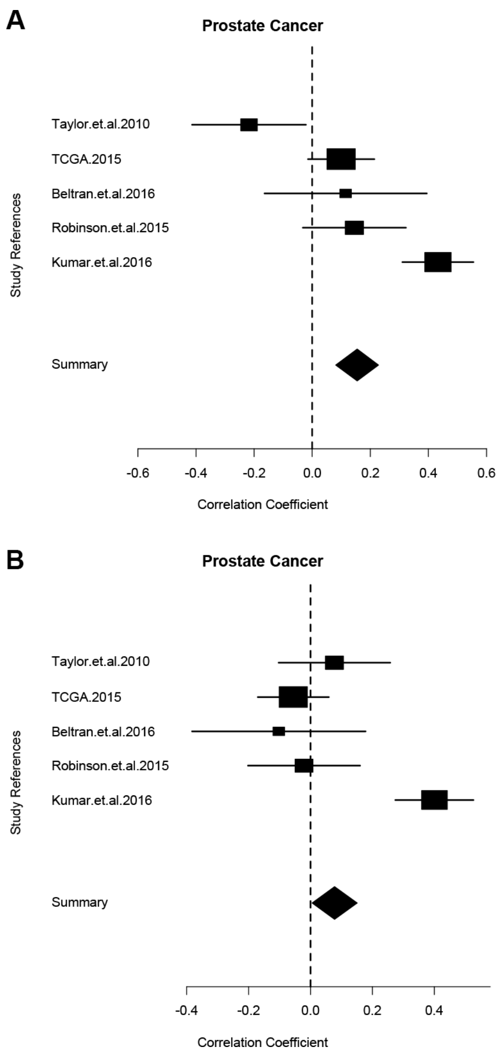

Supplementary Figure S4: *DNAH8* expression positively associated with AR-targeted gene *NKX3.1* and *FKBP5* expression in prostate cancer. From publically available database, we chose cohorts that contain over 100 patient cases to evaluate gene mRNA expression. Meta-analysis from 1476 prostate cancer cases showed a positive correlation coefficient in (**A**) *DNAH8-NKX3*.1(correlation coefficient 0.15, upper limit 0.23, lower limit 0.08, $p < 0.05$) and (**B**) *DNAH8-FKBP5* expression in prostate cancer (correlation coefficient 0.08, upper limit 0.15, lower limit 0.01, $p < 0.05$).

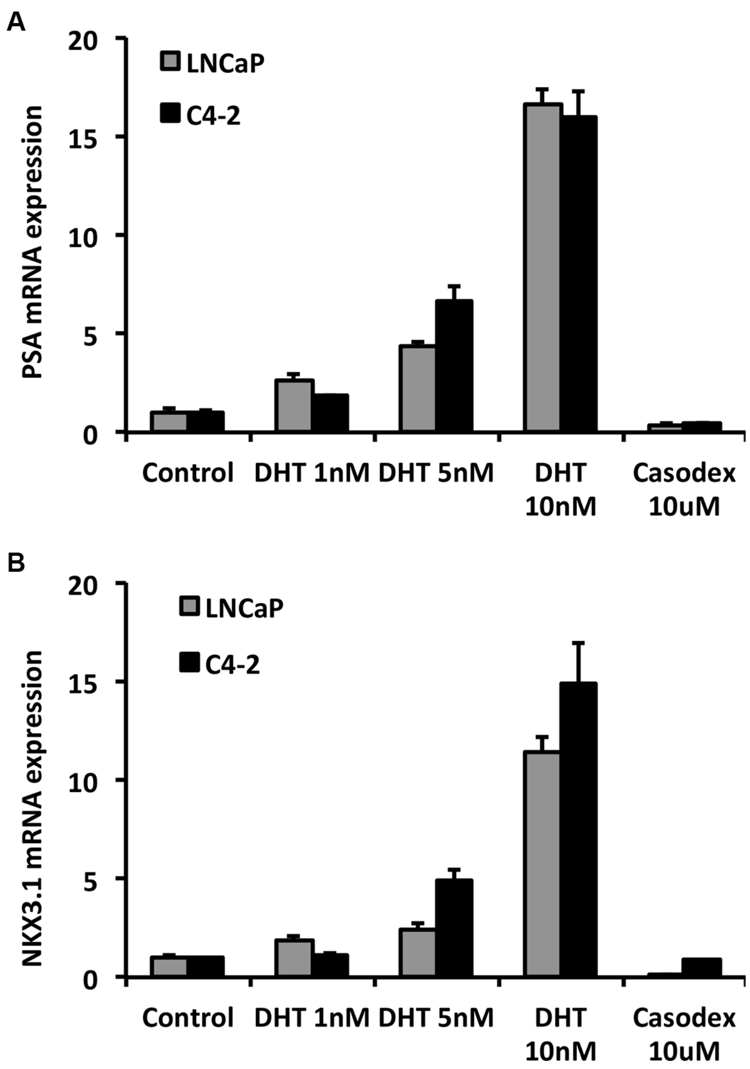

Supplementary Figure S5: AR promotes PSA and NKX3.1 expression in prostate cancer cells. LNCaP and C4-2 cells were treated as above with vehicle, DHT, or the AR antagonist Casodex (10 μM). After 48 hr, cell RNA was harvested for q-RT-PCR to measure PSA and NKX3.1 expression levels.

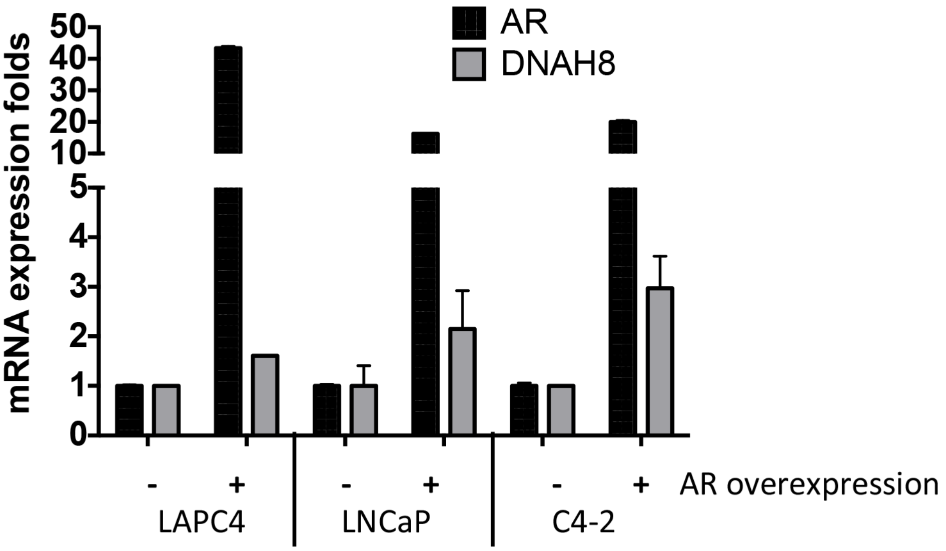

Supplementary Figure S6: AR overexpression in prostate cancer cell lines increased *DNAH8* expression. three different prostate cancer cells lines were transfected with wildtype AR. AR and DNAH8 mRNA were quantified by real-time PCR.

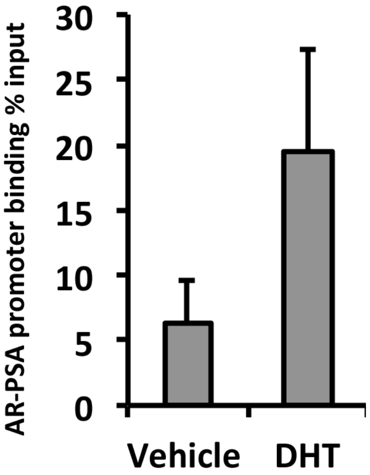

Supplementary Figure S7: DHT promoted AR binding to the *PSA* promoter. C4-2 cells cultured in complete media were treated with vehicle or 10 nM DHT for 24 hours. A ChIP assay for AR was performed. Primer pairs that span the ARE region of the *PSA* promoter were used to assess AR recruitment to the *PSA* promoter ($p = 0.02$).

Supplementary Table S1: DNAH8 siRNA sequence

DNAH8 siRNA 1
Sense: GGAAGAACUUACAUGUUGUtt
Antisense: ACAACAUGUAAGUUCUUCCtt
DNAH8 siRNA 2
Sense: CUACUUCCUUUCAGACUAUtt
Antisense: AUAGUCUGAAAGGAAGUAGtt
DNAH8 siRNA 3
Sense: GGAGAGUCAUUAAAGUUGAtt
Antisense: UCAACUUUAAUGACUCUCCtt

Supplementary Table S2: ChIP primers

**The *DNAH8* promoter primers**		
1F	Forward primer	AGAGCGGCTGGTGAGTACTTG
1R	Reverse primer	AAAACGGGGAGTGGGTGTGAA
2F	Forward primer	GGACTCCCAACCGTCAGC
2R	Reverse primer	GGACTGAACTGTAGCGTGGAT
3F	Forward primer	CTCGGTGGGTGCATCCTTTA
3R	Reverse primer	CGACCAAGTACTCACCAGCC
4F	Forward primer	CACGTATACGCGGGAATCCTCC
4R	Reverse primer	CGGAACGAGGCTGACGGTT
5F	Forward primer	TGAACACACTGACTCACCAGG
5R	Reverse primer	AATAAAGGATGCACCCACCGA
6F	Forward primer	CAGGCAGTCACAGCAAAAGC
6R	Reverse primer	TTGGGAGGATTCCCGCGT
7F	Forward primer	AAACGGACCGCTCACATCAT
7R	Reverse primer	TGACCTTTACAGTTTCTGAAGTTAC
8F	Forward primer	CTGGAAGAATGCCCCTGAAGA
8R	Reverse primer	GTGAGCGGTCCGTTTTTAGT
9F	Forward primer	CTTGCATTTCGGCACAGTCA
9R	Reverse primer	CCAGGGGCTGCACTTAGTC
10F	Forward primer	ACTGGGAGGCAGGTAAAGAATC
10R	Reverse primer	AAGCTTATGGGAGCAAGGGA
11F	Forward primer	AGTATCATATGGGAGGCCGGT
11R	Reverse primer	CTTTAGGCAACTCAGCAGGC
12F	Forward primer	GTATAAAACCTGCCCCAGCTC
12R	Reverse primer	TTCTTTACCTGCCTCCCAGTC
13F	Forward primer	GGATCACGGTGCTACAAACCTA
13R	Reverse primer	TGCTCTACCTCTTGTCCATCG
14F	Forward primer	AGGCATTCTCAGGGTTGCTA
14R	Reverse primer	TTCCTGTCCCTATGCTGTCTC
15F	Forward primer	CTCAGGATCGTGGGCACTTA
15R	Reverse primer	GCTGTATTAGGTTTGTAGCACCG
16F	Forward primer	ACAGGACTCAGTTTCCCTGAT
16R	Reverse primer	CCACAGAGTTCTCTTGAGGCTT
**The *PSA* promoter primers**		
1F	Forward primer	AGGGATCAGGGAGTCTCACA
1R	Reverse primer	GCTAGCACTTGCTGTTCTGC
**Real-time PCR primers**		
RPL19-F: CACAAGCTGAAGGCAGACAA<br>RPL19-R: GCGTGCTTCCTTGGTCTTAG<br>PSA-F: CCAAGTTCATGCTGTGTGCT<br>PSA-R: GCACACCATTACAGACAAGTGG<br>NKX3.1-F: AGAAAGGCACTTGGGGTCTT<br>NKX3.1-R: TTTGGGGAAGCCTTGGAA<br>DNAH8-F: TGCCCATCTCTGGAAGCATT<br>DNAH8-R: TTATCCGGGGCTGCATTGT		
